# Early‐Onset Posterior Reversible Encephalopathy Syndrome at Reduced‐Dose Sunitinib in Metastatic Papillary Renal Cell Carcinoma: A Case Report

**DOI:** 10.1155/carm/7058556

**Published:** 2026-05-04

**Authors:** Ferit Aslan, Burcu Erkılıç, Elif Günaydın, Onur Serdar Gençler, Hakan Taban

**Affiliations:** ^1^ Department of Medical Oncology, Yüksek İhtisas University Medicalpark Batıkent Hospital, Ankara, Türkiye; ^2^ Department of İnternal Medicine, Yüksek İhtisas Unıversity Medicalpark Batıkent Hospital, Ankara, Türkiye; ^3^ Department of Radiology, Yüksek İhtisas University Medicalpark Batıkent Hospital, Ankara, Türkiye; ^4^ Department of Neurology, Ankara Bilkent City Hospital, Ankara, Türkiye

**Keywords:** case report, posterior reversible encephalopathy syndrome, PRES, renal cell carcinoma, sunitinib, tyrosine kinase inhibitor, VEGF

## Abstract

Posterior reversible encephalopathy syndrome (PRES) is a rare but potentially life‐threatening neurological complication associated with vascular endothelial growth factor (VEGF)‐targeted tyrosine kinase inhibitors. Although it is typically reported after prolonged exposure and at standard or high doses, early onset at reduced dosing may occur and requires prompt recognition. We report a 64‐year‐old woman with metastatic papillary renal cell carcinoma who developed PRES after two months of treatment with reduced‐dose sunitinib (37.5 mg daily). She presented with severe headache, nausea, vomiting, impaired consciousness, and reduced mobility, accompanied by acute hypertension (170/100 mmHg). Brain magnetic resonance imaging demonstrated bilateral vasogenic edema without diffusion restriction, consistent with PRES, and electroencephalography revealed generalized background slowing indicative of encephalopathy. Sunitinib was immediately discontinued, and the patient was treated with corticosteroids, antiepileptic therapy, and antihypertensive agents, resulting in rapid clinical improvement. Follow‐up imaging confirmed complete radiological resolution. Subsequent treatment with nivolumab and axitinib achieved limited disease control, and the overall survival was 11 months. This case highlights that PRES can develop early and at reduced doses of sunitinib, may present with severe neurological impairment, and can be supported by electroencephalographic findings. Early recognition and prompt discontinuation of the offending agent are essential to prevent irreversible neurological damage.

## 1. Introduction

Posterior reversible encephalopathy syndrome (PRES) is an acute neurological disorder characterized by headache, seizures, visual disturbances, and altered mental status, typically associated with radiological findings of subcortical vasogenic edema [1]. Common predisposing factors include acute hypertension, renal failure, eclampsia, autoimmune disease, sepsis, and exposure to immunosuppressive or antiangiogenic agents [1, 2]. Epidemiological studies suggest that PRES occurs more frequently in women, with a median age of approximately 56 years [2]. Although often reversible, delayed recognition may lead to irreversible injury or death [1].

Among targeted therapies, VEGF receptor tyrosine kinase inhibitors (TKIs) such as sunitinib, pazopanib, sorafenib, regorafenib, axitinib, cabozantinib, lenvatinib, and tivozanib have been strongly implicated in PRES [2, 3]. These drugs are primarily metabolized through CYP3A4 and transported by ATP‐binding cassette transporters, with plasma concentrations closely linked to efficacy and toxicity [4, 5]. The proposed mechanism of TKI‐induced PRES involves VEGF inhibition, resulting in decreased nitric oxide bioavailability, endothelial dysfunction, impaired autoregulation, and disruption of the blood–brain barrier [1, 2].

Here, we present a case of sunitinib‐associated PRES in a patient with metastatic papillary renal cell carcinoma (RCC). This report highlights the diagnostic challenges, the clinical management, and the therapeutic implications of this rare but potentially severe complication.

## 2. Case Report

A 64‐year‐old woman with a history of papillary RCC presented to our clinic with progressive weight loss, anorexia, and worsening bone pain. She had previously undergone radical nephrectomy in the setting of synchronous metastatic disease for both diagnostic confirmation and symptom control; histopathological evaluation revealed a 7.5 cm papillary RCC, Grade 2, staged as T4N1M1. According to IMDC criteria, the patient had poor‐risk features, including anemia, a time interval of less than one year from diagnosis to initiation of systemic therapy, and reduced performance status, all of which were present prior to treatment initiation. Prognostically, she was classified as *poor risk* according to the International Metastatic RCC Database Consortium (IMDC) criteria and *intermediate risk* by the Memorial Sloan‐Kettering Cancer Center (MSKCC) model. A staging PET‐CT demonstrated extensive nodal, pulmonary, adrenal, and osseous metastases, highlighting the aggressive nature of her disease. The patient had no known history of chronic hypertension, cardiovascular disease, or prior neurological disorders. Baseline renal function was within acceptable limits for systemic therapy, and no other significant comorbidities were identified. The patient had no significant blood pressure elevations documented during earlier treatment cycles. The marked hypertension observed at presentation was therefore considered an acute event in the context of PRES.

Sunitinib was selected as first‐line therapy due to limited access to immune checkpoint inhibitor‐based combination regimens at the time of treatment initiation, as these therapies were not reimbursed by the national healthcare system, making sunitinib a necessary and practical choice in routine clinical practice. Given her poor‐risk profile, systemic therapy with sunitinib (50 mg daily, 2‐weeks‐on/1‐week‐off schedule) was initiated in combination with zoledronic acid for skeletal support. During the initial cycles, the patient developed several treatment‐related toxicities, including Grade 2 mucositis, stomatitis, thrombocytopenia, hyponatremia, and hypoalbuminemia. Owing to intolerance, the sunitinib dose was reduced to 37.5 mg daily on the same schedule.

After approximately two months of treatment, the patient acutely developed a pulsatile headache accompanied by nausea, vomiting, impaired consciousness, and reduced mobility. On admission, she appeared drowsy, responded inconsistently to commands, and exhibited weakness in the lower extremities. Her blood pressure was markedly elevated at 170/100 mmHg, despite no prior history of uncontrolled hypertension.

Neurological investigations were promptly performed. Brain magnetic resonance imaging (MRI) revealed bilateral vasogenic edema involving both cerebellar and cerebral hemispheres, with confluent hyperintensities on fluid‐attenuated inversion recovery (FLAIR) and T2‐weighted images, elevated apparent diffusion coefficient (ADC) values, and no diffusion restriction on diffusion‐weighted imaging (DWI) (Figures 1 and 2) These findings were characteristic of PRES. An electroencephalogram (EEG) performed at presentation showed generalized background slowing with diffuse theta–delta activity, consistent with encephalopathy (Figure 3). Additional diagnostic evaluations were performed in consultation with a neurology specialist to systematically exclude alternative neurological etiologies, including metastatic disease, infectious causes, and metabolic disturbances, all of which were unremarkable.

FIGURE 1Brain MRI at the onset of symptoms demonstrating bilateral vasogenic edema involving the cerebellar and cerebral hemispheres. Increased and confluent signal intensity is observed on fluid‐attenuated inversion recovery (FLAIR) (a) and T2‐weighted imaging (b). No diffusion restriction is observed on diffusion‐weighted imaging (DWI) (c). Apparent diffusion coefficient (ADC) mapping (d) shows increased signal in the cortical and subcortical regions, consistent with vasogenic edema and characteristic of posterior reversible encephalopathy syndrome (PRES). MRI = magnetic resonance imaging. A, MRI T1 FLAIR, B, MRI T2, C, DWI, E, ADC, respectively.

**Figure:**
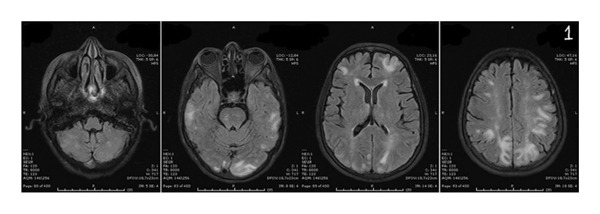
(a)

**Figure:**
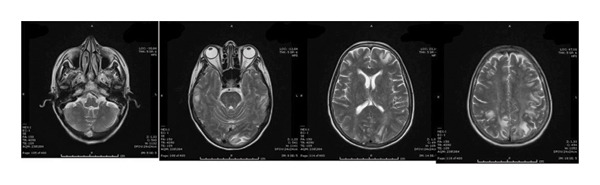
(b)

**Figure:**
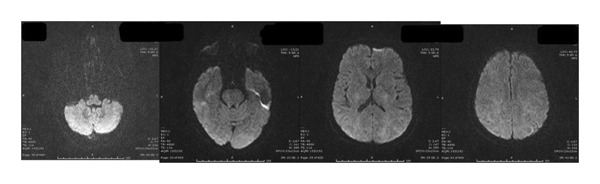
(c)

**Figure:**
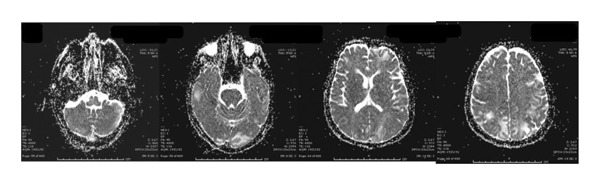
(d)

FIGURE 2The follow‐up brain MRI‐scans (FLAIR) of the brain during 8 days (a) and 8 weeks (b) after the discontinuation of sunitinib, the signal intensity had decreased. The lesions had disappeared 9 months after presentation of RPLS (c).

**Figure:**
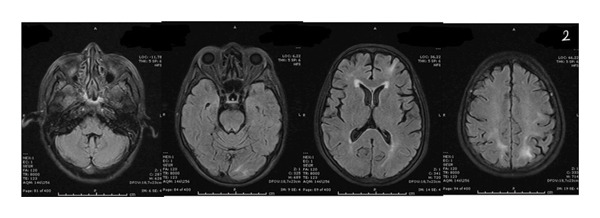
(a)

**Figure:**
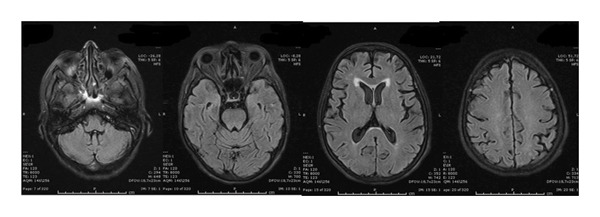
(b)

**Figure:**
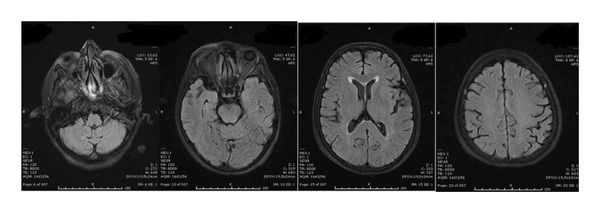
(c)

FIGURE 3Electroencephalography (EEG) recordings of the patient. (a) EEG at presentation demonstrating generalized background slowing with diffuse theta–delta activity, consistent with encephalopathy. (b) Follow‐up EEG showing restoration of normal alpha background activity with only minimal residual slowing, indicating significant clinical and electrophysiological improvement.

**Figure:**
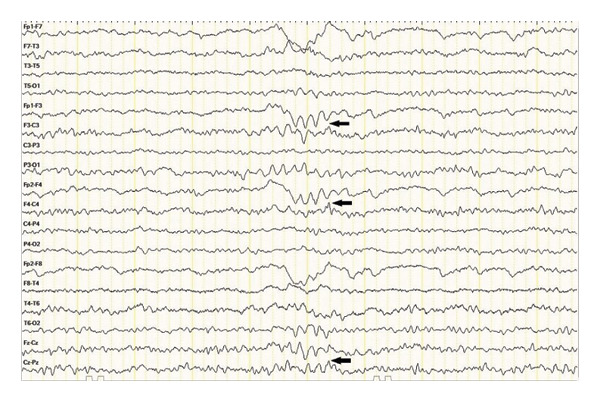
(a)

**Figure:**
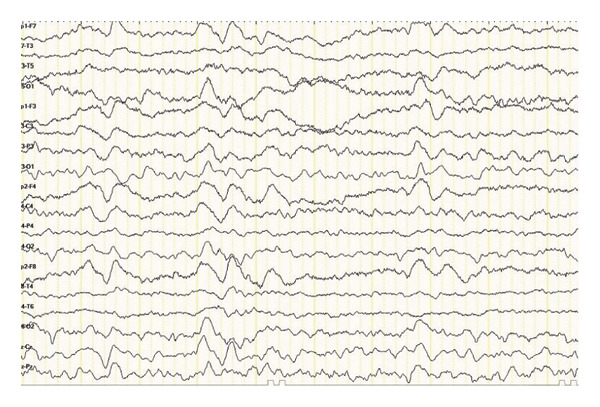
(b)

Sunitinib was immediately and permanently discontinued. The patient was started on dexamethasone (4 mg four times daily) for cerebral edema, valproic acid (1000 mg/day) for seizure prophylaxis, and antihypertensive therapy with benidipine (4 mg daily) and ramipril (5 mg daily). Her clinical condition improved rapidly: within days, her level of consciousness normalized, and mobility was restored. A follow‐up EEG performed eight days later demonstrated significant improvement, with restoration of normal alpha background activity and only intermittent bilateral fronto‐central theta discharges. Serial MRI scans confirmed progressive resolution of the cerebral edema over the following weeks, and complete disappearance of the lesions was observed nine months after the initial presentation.

Following recovery from PRES, second‐line treatment with nivolumab was initiated but resulted in a progression‐free survival of only three months. Third‐line axitinib was subsequently administered, also achieving a three‐month progression‐free interval. Ultimately, the patient’s total overall survival from the time of metastatic diagnosis was 11 months.

This case illustrates the acute and dramatic presentation of sunitinib‐associated PRES, even at a reduced dose and after a relatively short exposure period. It also emphasizes the reversibility of this potentially life‐threatening condition when promptly recognized and appropriately managed.

## 3. Discussion

PRES is increasingly recognized as a neurological complication of VEGF‐targeted TKIs. The pathophysiology involves endothelial dysfunction, impaired autoregulation, and blood–brain barrier disruption, with acute hypertension as a common precipitating factor, although PRES may also occur in normotensive patients [1, 2]. Rare but severe toxicities related to anti‐VEGFR therapies continue to be reported, highlighting the broad spectrum of potential adverse effects associated with these agents [6].

Several cases of sunitinib‐associated PRES have been published, typically occurring months after therapy initiation and at standard daily doses of 50 mg. Most patients experienced favorable neurological recovery following discontinuation of the drug and supportive care [3–5]. For example, Fukui et al. reported a case developing after six months of treatment with seizures and headache [3]. Saraceno et al. described PRES after prolonged low‐dose therapy, with visual disturbance as the primary manifestation [4]. Liu et al. documented a patient with gastrointestinal stromal tumor who developed PRES after several months of standard‐dose therapy [5]. These reports suggest variability in the timing and dosing associated with PRES development, though most cases occurred at higher exposure levels.

In addition to previously cited reports, several cases of VEGF‐targeted TKI‐associated PRES have been described, demonstrating considerable variability in clinical presentation [2–5]. Most reported cases occurred after prolonged exposure and at standard dosing, frequently presenting with seizures, visual disturbances, or altered mental status [3–5]. In contrast, early‐onset PRES and cases occurring at reduced doses appear to be less commonly reported [4]. Compared with these reports, our case is notable for the rapid onset of symptoms within two months of therapy initiation despite dose reduction, as well as the severity of neurological impairment at presentation. Furthermore, while radiological findings are well characterized in the literature, electroencephalographic abnormalities have been less frequently emphasized [2]. The presence of diffuse slowing on EEG in our patient, followed by normalization after treatment, supports its potential role as an adjunctive diagnostic and monitoring tool. These findings suggest that PRES associated with sunitinib may occur earlier and at lower exposure levels than previously recognized, underscoring the need for heightened clinical vigilance. Similarly, Rifino et al. reported a case of sunitinib‐associated PRES in a patient with gastrointestinal stromal tumor, further supporting the association between VEGF‐targeted therapy and this neurological complication [7].

Our case differs from previously reported cases in several ways. First, PRES developed after only two months of therapy and at a reduced sunitinib dose (37.5 mg), demonstrating that even lower doses may be sufficient to trigger the syndrome. Second, the patient exhibited a severe clinical presentation, including altered consciousness and hypertension (170/100 mmHg), requiring urgent intervention. Third, EEG findings demonstrated diffuse slowing at onset and improvement upon follow‐up. Although rarely described in PRES, EEG may serve as a valuable adjunctive diagnostic tool in patients with unexplained encephalopathy. Finally, while rechallenge with TKIs has been reported in select cases [8], sunitinib was permanently discontinued in this patient. Sequential therapy with nivolumab and axitinib was attempted, but survival was ultimately limited, reflecting the poor‐risk biology of metastatic papillary RCC.

Importantly, PRES remains a diagnosis of exclusion, and alternative neurological etiologies were considered in our patient. Paraneoplastic and immune‐mediated processes were deemed less likely based on the acute onset of symptoms, the presence of severe hypertension, and the characteristic imaging pattern of vasogenic edema without diffusion restriction. Furthermore, the rapid and complete clinical and radiological recovery following discontinuation of sunitinib and supportive treatment strongly supported the diagnosis of PRES. In contrast, paraneoplastic neurological syndromes typically follow a more subacute or progressive course and are less likely to demonstrate such prompt reversibility. In our case, this diagnostic approach was supported by multidisciplinary evaluation, including neurological consultation and additional investigations, all of which were unremarkable and did not suggest an alternative etiology.

From a clinical perspective, this case underscores key lessons. Continuous blood pressure monitoring and early neuroimaging should be prioritized in patients receiving VEGF‐targeted therapies. EEG, although nonspecific, may provide diagnostic support in complex presentations. Decisions regarding rechallenge with TKIs must be carefully individualized, balancing oncological efficacy against the risk of recurrent PRES. Moreover, therapeutic drug monitoring may provide future opportunities to identify patients at higher risk [9]. In this context, pharmacodynamic studies, such as the analysis of peripheral blood mononuclear cells, have been proposed as potential biomarkers for treatment response and toxicity in patients receiving sunitinib [10].

In our patient, corticosteroid therapy was initiated to manage significant cerebral edema in the context of acute neurological deterioration, including impaired consciousness, in association with extensive vasogenic edema on neuroimaging. Although corticosteroids are not routinely indicated in the management of PRES, they may be considered in selected cases for symptomatic control. Importantly, the primary intervention remains discontinuation of the offending agent and appropriate blood pressure management.

In summary, PRES can occur early and at reduced sunitinib doses, may present with severe neurological symptoms, and can be supported by EEG findings. Early recognition and prompt intervention are essential to prevent irreversible outcomes.

## Funding

No funding was received for this manuscript.

## Consent

Written informed consent was obtained from the patient for publication of this case report and any accompanying images.

## Conflicts of Interest

The authors declare no conflicts of interest.

## Data Availability

All data supporting the findings of this case, including clinical records and imaging data, are available from the corresponding author upon reasonable request for editorial and peer‐review purposes. Data will be shared in accordance with institutional policies and patient confidentiality requirements.

## References

[1] Fugate J. E. and Rabinstein A. A. , Posterior Reversible Encephalopathy Syndrome: Clinical and Radiological Manifestations, Pathophysiology, and Outstanding Questions, Lancet Neurology. (2015) 14, no. 9, 914–925, 10.1016/s1474-4422(15)00111-8, 2-s2.0-84939511933. 26184985

[2] Tlemsani C. , Mir O. , Boudou-Rouquette P. et al., Posterior Reversible Encephalopathy Syndrome Induced by Anti-VEGF Agents, Targeted Oncology. (2011) 6, no. 4, 253–258, 10.1007/s11523-011-0201-x, 2-s2.0-84856029091. 22090260

[3] Fukui S. , Toyoshima Y. , Inoue T. , Kagebayashi Y. , and Samma S. , Reversible Posterior Leukoencephalopathy Syndrome After Restart of Sunitinib Therapy for Metastatic RCC, Case Reports in Medicine. (2016) 2016, 10.1155/2016/6852951, 2-s2.0-85016860167. PMC5067324 27795711

[4] Saraceno L. , Ricigliano V. A. G. , Cavalli M. , and Meola G. , PRES After Long-Term Treatment With Low-Dose Sunitinib: A Case Report, Neurological Sciences. (2017) 38, no. 5, 903–906. 28224329 10.1007/s10072-017-2851-7

[5] Liu X. , Hong W. , Wang L. , and Zhang Z. , Sunitinib-Associated Posterior Reversible Encephalopathy Syndrome in a Patient With a Gastrointestinal Stromal Tumor, Asian Journal of Surgery. (2022) 45, no. 6, 1964–1965, 10.1016/j.asjsur.2022.04.034. 35525690

[6] Catalano F. , Rebuzzi S. E. , Murianni V. et al., Rare Anti-VEGFR Therapy-Induced Toxicity and Long-Term Response to Immunotherapy in a Rare Non-Clear Cell Renal Cell Carcinoma Patient, Anti-Cancer Drugs. (January 1 2022) 33, no. 1, e724–e729, 10.1097/CAD.0000000000001152. 34261919

[7] Rifino N. , Mantero V. , Filizzolo M. G. et al., Sunitinib Associated Posterior Reversible Encephalopathy Syndrome in a Patient Treated for GIST, Acta Neurologica Belgica. (August 2020) 120, no. 4, 995–997, 10.1007/s13760-020-01367-6. 32372399

[8] Deguchi S. , Mitsuya K. , Nakasu Y. et al., Posterior Reversible Encephalopathy Syndrome Induced by Pazopanib in a Patient With Soft-Tissue Sarcoma: Case Report and Review of the Literature, Investigational New Drugs. (2018) 36, no. 2, 346–349, 10.1007/s10637-017-0521-5, 2-s2.0-85032014498. 29067537 PMC5869870

[9] Puisset F. , Mseddi M. , Mourey L. et al., Therapeutic Drug Monitoring of Tyrosine Kinase Inhibitors in the Treatment of Advanced Renal Cancer, Cancers (Basel). (2023) 15, no. 1, 10.3390/cancers15010313. PMC9818258 36612311

[10] Noé G. , Bellesoeur A. , Thomas-Schoemann A. et al., Clinical and Kinomic Analysis Identifies Peripheral Blood Mononuclear Cells as a Potential Pharmacodynamic Biomarker in Metastatic Renal Cell Carcinoma Patients Treated With Sunitinib, Oncotarget. (October 11 2016) 7, no. 41, 67507–67520, 10.18632/oncotarget.11686, 2-s2.0-84994000514. 27589830 PMC5341893

